# Unravelling the Added Value of Urinary Stone Cultures Towards Infectious Complications Following Treatment of Renal Stones

**DOI:** 10.3390/antibiotics15010052

**Published:** 2026-01-04

**Authors:** A. V. B. Krishnakanth, Padmaraj Hegde, Arun Chawla, Sunil Bhaskhara Pillai, Pilar Laguna, Jean de la Rosette

**Affiliations:** 1 Department of Urology and Renal Transplant, Kasturba Medical College, Manipal, Manipal Academy of Higher Education, Manipal 576104, India; krishnakanth002@gmail.com (A.V.B.K.); padmaraj.hegde@manipal.edu (P.H.); sunil.pillai@manipal.edu (S.B.P.); 2 Department of Urology, Istanbul Medipol Mega University Hospital, Istanbul 34214, Türkiye; m.p.lagunapes@gmail.com; 3 International School of Medicine, Istanbul Medipol University, Istanbul 34810, Türkiye; j.j.delarosette@gmail.com; 4 Department of Urology, Bashkir State Medical University, Ufa 450008, Russia

**Keywords:** stone culture, infection, urolithiasis, complication, PCNL

## Abstract

**Aim:** To explore the association between urinary stone cultures and infectious complications following PCNL. **Materials and Methods:** An observational case–control study was conducted in patients undergoing PCNL. The assessment included demographic parameters, medical history, urinalysis, and urine culture and blood testing. Pre-operatively, urinary stone samples were collected for cultures. Post-operatively, patients were observed for infectious complications such as fever and/or SIRS. Patients were divided into two groups based on the presence or absence of infected renal calculi. Patient characteristics, stone factors, and intra-operative and post-operative findings were studied in relation to stone culture. Descriptive statistics was used to present the data and the SPSS software was used for analysis. **Results:** From December 2023 to March 2025, a total of 126 patients were included in the study. A total of 16 patients (12.6%) had a positive stone culture. Statistical significance was found upon the comparison of stone culture with gender (*p* = 0.046), chronic kidney disease (*p* = 0.002), pre-operative urine culture (*p* = 0.001), pre-operative haemoglobin (g/dL) (<0.001), pre-operative S. creatinine (mg/dL) (*p* = 0.038), stone volume (mm^3^) (*p* = 0.012), CROES score (*p* = 0.023), SIRS (*p* = 0.001), and AKI (*p* = 0.021). **Conclusions:** Infected renal calculi identified by positive stone cultures were strongly associated with infective complications such as fever and SIRS following PCNL. *E. Coli* was the dominant bacteria present in both bladder urine and renal stone culture. The occurrence of infectious complications despite the administration of pre-operative antibiotics highlights the antibiotic resistance patterns noted among the cultured bacteria. The pre-operative factors identified to be associated with a positive stone culture could potentially be used for predicting infected stones, thereby improving outcomes.

## 1. Introduction

Infectious complications represent the most frequent complication following endourological procedures for renal stone clearance and include fever, systemic inflammatory response syndrome (SIRS), and sepsis [1]. Various patient characteristics, stone aspects, and peri-operative factors influence the chances of infection following PCNL. One of the most important risk factors among them is the presence of an infection stone. Infection stones comprise 5–15% of all stones [2,3]. The origin of an infection stone might be de novo or due to the infection of a pre-existing renal stone. Infection stones have been historically defined as magnesium ammonium phosphate or struvite stones formed by urease-producing organisms [4]. Non-struvite stones may also be infected secondarily by a urinary tract infection [5]. Multiple variations exist in defining infection stones due to the presence of various criteria, such as large stone size, the presence of bacteria within the stone, the presence of bacteria on the surface of the stone, and the presence of the stone in an infected urinary system. Since the majority of infection stones present with a larger size and require complete stone removal to clear the infective nidus and prevent recurrent infection, procedures with the highest rates of stone clearance, such as PCNL, are preferred. Despite the standardizations and advancements in antibiotic protocols, the high incidence of infection stones in India is alarming, which in turn could have an effect on the infective complications following treatment with surgical procedures [6].

The primary aim of the present study is to understand the associations between stone culture and infectious complications following PCNL. Secondary aims include the exploration of the relations between urinary stone cultures with antibiotic sensitivity patterns, urine culture, peri-operative renal function, and stone composition.

## 2. Results

**Figure 1:**
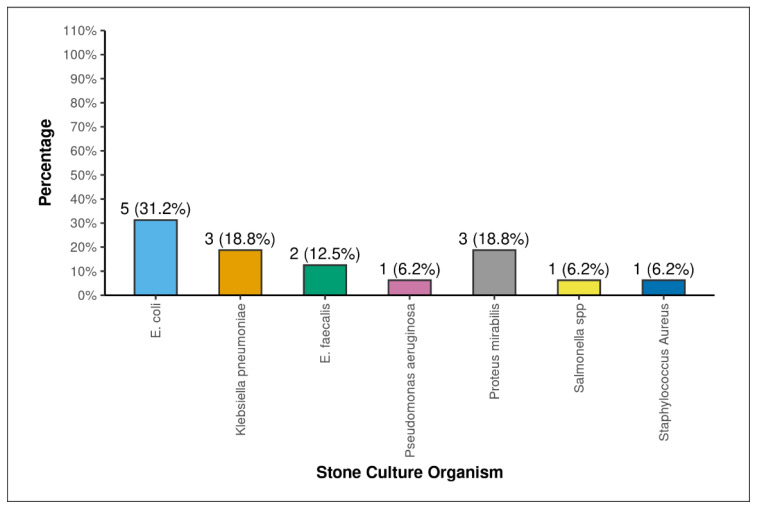
Distribution of stone culture organisms.

**Figure 2:**
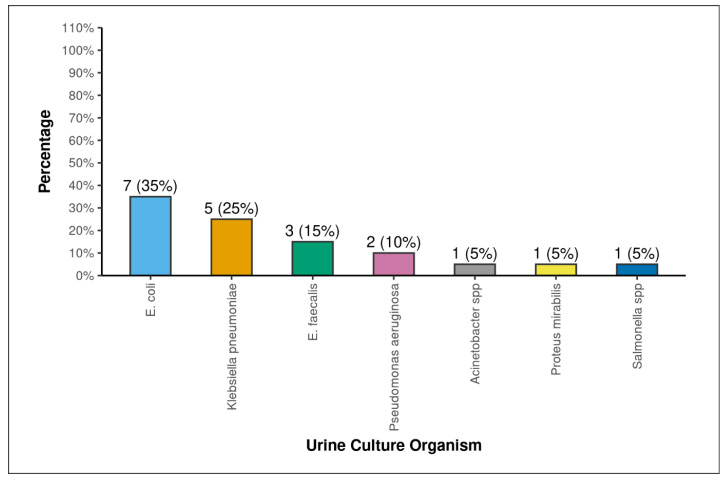
Distribution of urine culture organisms.

A total of 126 patients were included in the study, consisting of 83 males and 43 females, and with a mean age of 50.29 +/− 12.28 years. The demographic, patient factors, stone factors, and intra-operative and post-operative factors of the study population are shown in Table 1, Table 2, Table 3 and Table 4. Out of the 126 patients for whom renal stones were sent for culture sensitivity, 16 (12.6%) had positive stone cultures. The mean age of the patients was similar between the stone culture-positive (SCP) and stone culture-negative (SCN) groups (53.5 +/− 14.78 vs. 49.83 +/− 11.88, *p* = 0.355). Statistical significance was found in the comparisons of stone culture with gender (*p* = 0.046), chronic kidney disease (*p* = 0.002), pre-operative urine culture (*p* = 0.001), pre-operative haemoglobin (g/dL) (<0.001), pre-operative serum creatinine (mg/dL) (*p* = 0.038), staghorn (Mishra Classification) (*p* = 0.001), stone analysis: magnesium (*p* = 0.017), stone analysis: ammonia (*p* = 0.006), stone volume (mm^3^) (*p* = 0.012), CROES score (*p* = 0.023), energy source (*p* = 0.028), amplatz size (*p* = 0.045), type of PCNL (*p* = 0.037), residual stone fragments (*p* = 0.009), post-operative fever (*p* = 0.001), SIRS (*p* = 0.001), qSOFA score (*p* = 0.008), NEWS score (*p* = 0.001), NEWS clinical risk (Sepsis) (*p* = 0.001), post-operative serum creatinine (mg/dL) (*p* = 0.001), post-operative haemoglobin (g/dL) (*p* = 0.001), post-operative total leukocyte count (/mm^3^) (*p* = 0.001), hospital stay (*p* = 0.001), modified Clavien–Dindo classification (*p* = 0.001), and post-operative acute kidney injury (*p* = 0.021). The incidences of post-operative complications according to the modified Clavien classification score for PCNL was as follows: No Complication = 64 (50.8%), Grade 1 = 18 (14.3%), Grade 2 = 11 (17.5%), Grade 3A = 19 (15.1%), Grade 3B = 1 (0.7%), and Grade 4A = 2 (1.6%). No patients had Grade 4B or 5 complications. The various bacterial growths noted in the renal stone cultures are shown in Figure 1, and in Figure 2, the pre-operative urine cultures are presented. In both cultures, *E. Coli* is the dominant bacteria present. The antibiotic sensitivity and resistance of various organisms are shown in Table 5.

Univariate regression analysis between stone culture positivity and various variables is shown in Table 6. The presence of Systemic Inflammatory Response Syndrome (SIRS) was a powerful univariable predictor, increasing the odds of a positive stone culture by 95 times (OR = 95.00, 95% CI [17.37–1781.76], *p* < 0.001). In the univariable regression analysis, each one-point increase in the qSOFA Score was associated with a 3.24-fold increase in the odds of a positive stone culture (OR = 3.24, 95% CI (1.31–8.39), *p* = 0.011). Clinically, the univariable result remains important: a higher qSOFA score is a potent indicator of risk for a positive stone culture. Each one-point increase in the National Early Warning Score (NEWS) was associated with a 2.43-fold increase in the odds of a positive stone culture (OR = 2.43, 95% CI [1.77–3.74], *p* < 0.001). Clinically, the univariable result remains important: a higher NEWS score is a potent indicator of risk for a positive stone culture. The variables for Fever and NEWS Clinical Risk (Sepsis) produced statistically uninterpretable results in univariable regression analysis with extremely large odds ratios and non-significant *p*-values (e.g., *p* = 0.991).

No independent variable was statistically significant in the multivariable model. This statistical phenomenon is attributed to a combination of high multicollinearity between the predictors (SIRS, NEWS score, and qSOFA score all measure similar aspects of patient acuity).

Univariate logistic regression analysis was performed for statistically significant pre-operative predictors of positive stone culture on univariate analysis, and is shown in Table 7.

## 3. Discussion

The incidence of culture-proven infection stones in the present study is 12.6%, which corresponds to the global incidence [3,6,7]. The most-common organism cultured was *E. coli* (31.2%), followed by Proteus mirabilis (18.8%) and Klebsiella pneumoniae (18.8%). Since only rare species of *E. coli* produce urease, the growth of E.coli mostly signifies non-struvite secondarily infected renal calculi [5]. The present study also revealed that almost all the stones with E.coli growth were of non-struvite composition. Of the patients with growths of E.coli on their stone culture, 80% also had *E. coli* growth in their urine culture, which further confirms our suspicion of secondarily infected renal calculi.

In the present study, mid-stream urine culture samples (MSUCs) obtained prior to PCNL showed a growth in 15.87% of the patients. More than half of the patients with stone culture growth also exhibited growth in their mid-stream urine culture, and all of them showed growths of the same organism in both the stone and mid-stream urine cultures. This contradicts the finding of Mariappan et al., who stated in their study that bladder urine is a poor predictor of infection stones and showed a discrepancy between stone and bladder urine cultures in the organisms grown [8].

Although stone culture is the standard investigation, as used in our study for the confirmation of infection in a renal calculus, the presence of infection in renal pelvic or bladder urine should also prompt suspicion of infected renal calculi, even in the absence of demonstrable growth in the stone culture. The optimal collection of stone and urine samples is crucial for avoiding false results. The locus of bacteria differs between a primary infection stone and a secondarily infected stone. A stone sample obtained from the periphery of a primary infection stone might not be representative of the central core, where the nidus is expected to be. Similarly, a central core obtained from secondarily infected renal calculi may not be representative of the central core. In the present study, samples from both the periphery and the core were collected after fragmentation and were sent together for culturing, thereby effectively reducing the chances of missing any nidus of infection.

Females have a higher incidence of infection stones, and similar findings were also found in the present study, where positive stone culture incidence in females (20.9%) was higher when compared to that of males (8.4%) [9].

While the presence of risk factors such as diabetes and spinal injury, among others, have been associated with an increased risk of infection stone formation, it is difficult to explain the presence of infection stones in patients who lack such risk factors. The present study does not show any significant differences among comorbidities in the stone culture-positive and -negative groups apart from the increased prevalence of CKD in the stone culture positive-group. It is of interest to study why one stone is infected and the other one not. Increased stone volume, indiscriminant use of antibiotics, and altered or abnormal urinary microbioma could potentially play a role in the formation of infection stones, especially in patients without any established risk factors.

Infection stones cause recurrent and chronic urinary tract infections, along with the propensity to grow rapidly, thereby occupying the entire pelvi-calyceal system, resulting in the loss of renal function, and progressing to end-stage renal disease and kidney loss [10,11,12]. In the present study, 31.2% of patients with infected stones had chronic kidney disease and had a higher serum creatinine at the time of presentation compared to metabolic stones. Further research is required to determine whether the association between renal stones and chronic kidney disease is causal or due to the presence of similar risk factors. The chronic kidney disease patients in the present study were diagnosed with CKD stages 3, 4, or 5 with coexisting diabetes (55.6%), systemic hypertension (88.9%), and a history of recurrent UTIs. The low pre-operative haemoglobin noted in the stone culture-positive group is attributed to anaemia of chronic kidney disease.

**Figure 3:**
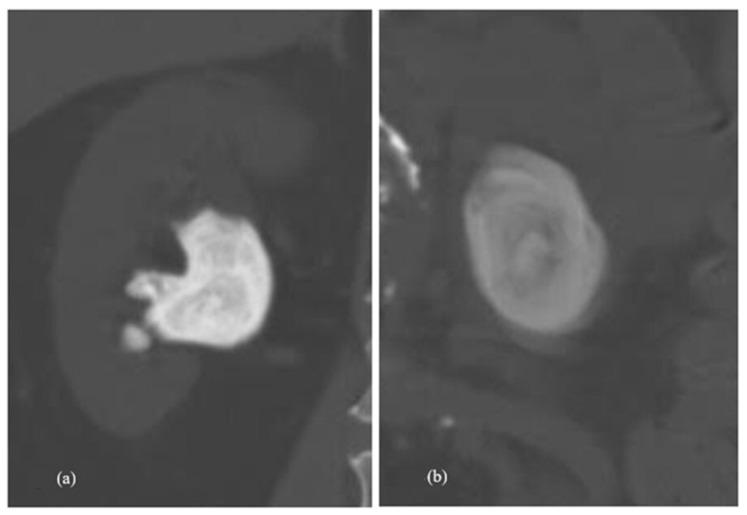
Computed tomography (bone window) of renal calculi. (**a**) Non-infected renal calculi and (**b**) infected renal calculi.

An important factor supporting the presence of infection stones is the stone size. Infection stones have the tendency to rapidly increase in size due to the formation of bacterial biofilms, the urinary environment, and crystallisation. In the present study, we found infected stones to have a larger stone volume when compared to non-infected stones, with the mean being almost twice as large as compared to non-infected stones. A study on the composition of infected stones by Dajaivi et al. [13] revealed that most of the infected stones in their study were of the oxalate type (55%), followed by the uric acid/urate (25%), calcium phosphate (20%), and struvite stone types (5%). Similarly, in the present study, we also noted higher rates of calcium stone compositions in culture-positive renal stones. Among the 16 patients with stone culture growth in our study, 8 (50%) had stones with mixed struvite compositions, 7 (43.7%) had calcium stones, and 1 (6.25%) had uric acid stones. No pure struvite stones were encountered. Infection stones have a lower Hounsfield unit on CT imaging compared to their counterparts, with decreasing HU from the core to the distal periphery of the stone [14]. In the present study, we noted comparatively lower Hounsfield units in the stone culture-positive group but failed to show a statistically significant difference. According to the present study, the review of patients with infected and non-infected stones revealed a central hypodense core in infection stones on the bone window of plain CTs, as shown in Figure 3, suggesting an infectious nest within the stone (which might be pure struvite), surrounded with mixed struvite forms. This finding might support the hypothesis of the core stone culture being more positive than the periphery, requiring zero stone residue and post-operative antibiotics for longer periods. Among the nephrolithometry scoring systems used in the study, the CROES score was found to be statistically significant in relation to stone culture, with a lower CROES score noted in the stone culture-positive group. Reported studies lack evidence in associating stone culture positivity with the size of the stone, the use of antibiotics, and altered microflora. The absence of urinary tract anomalies and the presence of stents/PCN/catheters rule out colonisation in the present study.

In the present study, the use of a larger Amplatz sheath in stones with large volumes facilitated timely removal along with complete stone clearance, which are both crucial to preventing infective complications and stone recurrence.

According to a systematic review by Falahatkar et al. [15], the prevalence rate of fever among patients undergoing PCNL is 9.5%. Although we found a higher incidence of fever in patients following PCNL (24.6%), all patients with a positive stone culture had fever, and the incidence of fever among patients who did not show any growth on stone culture was only 13.6%. In the present study, pre-operative leukocyte counts showed no significant difference between the stone culture-positive and -negative groups, but the post-operative total leukocyte count showed statistical significance. We noted higher complication rates in the stone culture-positive group according to the modified Clavien–Dindo classification for PCNL. Limited research is available on the relation between stone culture and AKI following PCNL. Sepsis is among the most important factors that contribute to post-operative acute kidney injury [16,17]. In the present study, we found that patients with infected stones are three times more prone to developing AKI following PCNL compared to patients with non-infected stones.

A randomised study showed that in patients with negative pre-operative urine cultures, a short course of pre-operative antibiotic prophylaxis does not influence the incidence of sepsis following PCNL [18]. Antibiotic sensitivity testing of the bacterial cultures obtained from renal stone and bladder urine in the present study revealed increased resistance to third-generation cephalosporins and fluoroquinolones, but most of the organisms were still sensitive to the carbapenem group. The emergence of antibiotic resistance could be attributed to the recurrent prior use of higher antibiotics in patients with a previous history of urolithiasis and appropriate surgical intervention. In the present study, 50% of the patients in the stone culture-positive group had a history of urolithiasis for which endourological intervention was performed. Although not statistically significant, this was still higher than that of the control group (culture-negative) with 27.3%. This might have contributed to the emergence of infection stones and antibiotic resistance due to previous treatment with antibiotics. The emergence of antibiotic-resistant microbial variants emphasises the importance of antimicrobial stewardship and the need for culture-guided antibiotic use rather than empirical antibiotics to reduce resistance and improve outcomes.

Given the larger size of the stone at presentation in patients with infection stones, it is highly likely that the patient might have been treated conservatively with analgesics and antibiotics prior to presentation at a tertiary care centre for definitive management. The increased occurrence of infection stones is also well recognised in India, which, along with the unrestricted usage of higher antibiotics as a first-line management tactic, could also play a role in the emergence of antibiotic resistance.

The antibiogram helped in deciding the appropriate antibiotic since nearly all the patients in the infection group developed SIRS despite administering third-generation cephalosporins peri-operatively. All the patients in the stone culture-positive group were treated with culture-sensitive antibiotic therapy for 1 month and recovered promptly with no further escalation of infectious complications. Complete stone removal is preferred in patients undergoing PCNL, as residual stones form a nidus for infection and stone recurrence [19]. Similarly, findings of a higher incidence of post-operative infective complications were noted in patients with residual fragments or incomplete removal during PCNL.

The incidence of SIRS in the present study was 23.8%, which was similar to other studies [20,21,22,23]. We also found that the presence of SIRS showed a strong association with the presence of an infection stone. The incidence of SIRS following PCNL was 93.75% in patients with positive stone cultures, whereas it was only 13.6% in the negative stone culture group. Despite this, the incidence of SIRS was twice that of the incidence of infected renal stones when considering the entire study population. Further research into the group of patients with negative stone cultures who developed SIRS revealed a significant association with the presence of chronic kidney disease and urinary tract infection, and almost 73.3% of these patients had positive pre-operative bladder urine cultures. On comparing SIRS-positive and SIRS-negative groups among patients with negative stone cultures, there were no significant differences noted in the stone and operative factors.

The present study highlights the increased risk of infectious complications in patients with positive stone cultures, which is in accordance with the literature [20,22,23,24,25]. Table 8 shows the findings of other studies on the association of stone culture and infectious complications following PCNL, along with the tools used to clinically define sepsis in their studies.

Even with advancements and improvements in health coverage, the occurrence of infected stones remains the same, accompanied by increasing antibiotic resistance. This subset of patients progress to sepsis post-operatively despite the initiation of antibiotics pre-operatively. It also reveals a higher incidence of acute kidney injury following PCNL among stone culture-positive patients.

## 4. Strengths and Limitations

The strength of the present study lies in its prospective nature. Well-established, standard protocols were used for stone collection and culture. An extensive study of the antibiotic sensitivity of the grown organisms brings to focus the alarming resistance patterns in the local community. Our study also highlights the association between infection stones and acute kidney injury following PCNL. The present study confirms earlier findings presented in the literature. In addition, our finding that mid-stream urine culture is not a poor predictor of infection stones does not align with the findings of previous studies. This study also showed the high probability of the presence and isolation of the same organism from both mid-stream urine and renal calculi in patients with infection stones. The presence of an indwelling DJ stent or a previous urological procedure did not significantly increase the incidence of infected renal calculi.

We acknowledge the study’s limitations due to the single-institute nature of the study, leading to a decrease in the size of the stone culture-positive group. Multi-institutional collaboration in the future would address this limitation. Despite strict protocols being instituted in the study, bias in sample collection or the washing of the stones during the PCNL may have potentially contributed to false-negative stone cultures. Furthermore, the present study utilises quantitative chemical analyses for stone analysis, which are inferior to the Fourier transform infrared (FTIR) and X-ray diffraction methods. The long-term sequelae of patients developing fever or urosepsis following PCNL were not studied.

## 5. Materials and Methods

A prospective observational case–control study was conducted from December 2023 to March 2025. Ethical approval was obtained from the institutional ethical committee (EC/NEW/INST/2022/KA/0042—IEC1: 316/2023) and registered with the Clinical Trials Registry of India (CTRI/2023/11/060307) before the study began.

**Figure 4:**
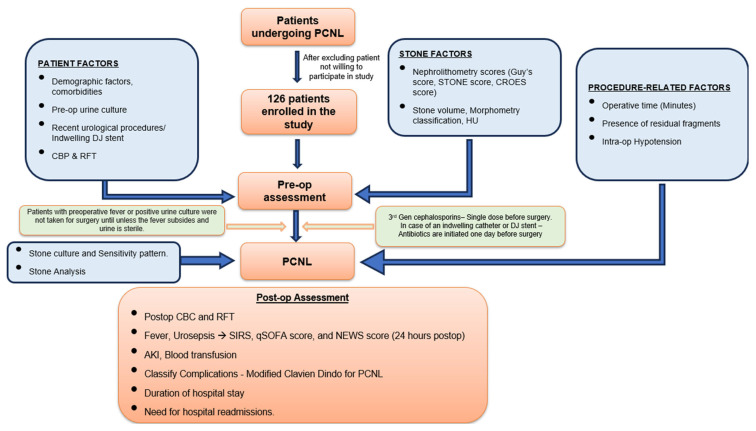
Flowchart of study timeline (RFT = renal function test, CBC = complete blood count, SIRS = systemic inflammatory response syndrome, qSOFA = quick SOFA, NEWS = national early warning score, and AKI = acute kidney injury).

**Figure 5:**
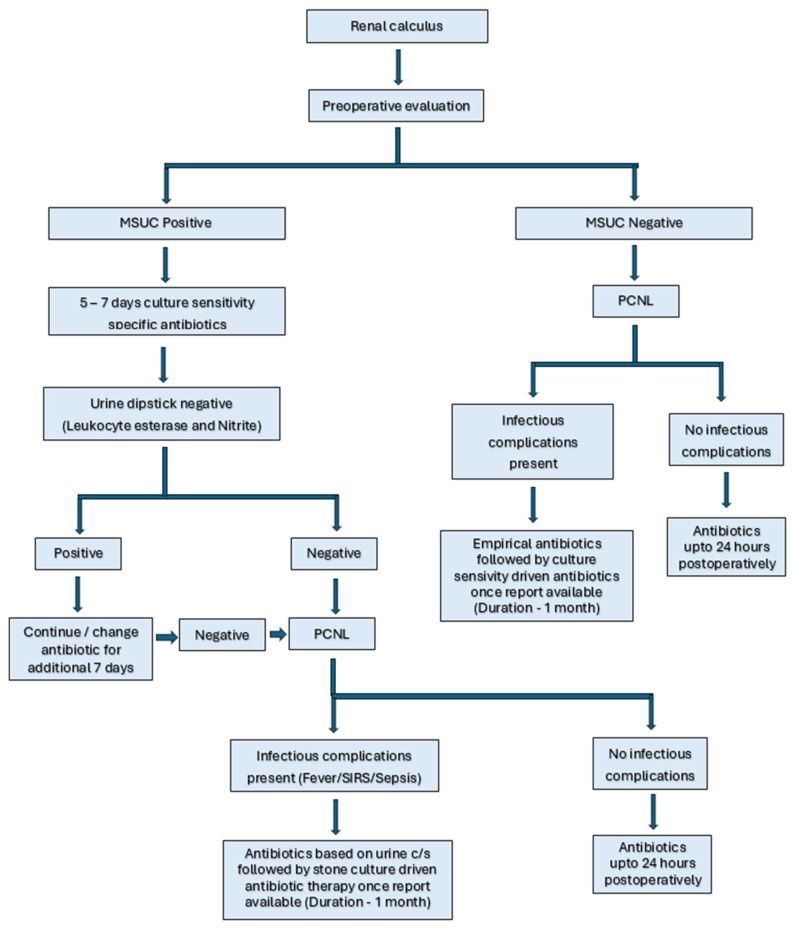
Flowchart of evaluation and management of renal stones in our study.

As per protocol, all renal stones planned for PCNL underwent non-contrast computed tomography KUB. Patients who met the inclusion and exclusion criteria were included in the study after informed consent was obtained. Patient factors such as demographics, comorbidities, recent uro-surgical history (within 3 months), routine blood panel, urine analysis, and mid-stream urine culture (MSUC) were collected pre-operatively. Patients with pre-operative positive MSUCs were treated with antibiotics for 5 to 7 days and taken for the procedure following a negative urine dipstick test for detecting infection. A flowcharts depicting the study methodology, evaluation and management of patients with renal stones in our study is provided in Figure 4 and Figure 5. Stone factors were recorded via NCCT KUB (size in terms of volume (AxBxCx0.167 × 3.14), laterality, site, Hounsfield value, hydronephrosis, staghorn stone, and nephrolithometry scores) pre-operatively. The Society for Foetal Urology (SFU) grading was used to grade hydronephrosis [31]. In our study, SFU Grade 1 was referred to as mild, grade 2 as moderate, and Grades 3 and 4 as gross. Antibiotic prophylaxis was administered as per protocol following institutional guidelines (third-generation cephalosporins −1 h before surgery). In our study, mini PCNL is defined as ≤22Fr dilatation, and standard PCNL as ≥24Fr dilatation. Operative factors were recorded, such as standard vs. mini PCNL, Amplatz size, tract length, number of punctures, site of puncture, the use of pneumatic lithotripter or LASER for clearance, operative time, intra-operative hypotension, and the presence of residual fragments.

Renal calculi samples obtained from the periphery and core of the renal calculi during PCNL were collected using the Nemoy and Stamey method, which is meant to wash off surface contaminants and culture bacteria within the stone [32]. Fragments are washed in 5 sequential bottles containing sterile normal saline, after which the contents were sent for stone culture and sensitivity testing. The calculi were crushed before being subjected to culture, and the crushed calculi core is cultured in blood agar and MacConkey agar. The growth of organisms and sensitivity was reported at 48 h. Patients with no growth in stone culture were labelled as the control group. The microbiologists involved in the study of microbial growth on the stone cultures were blinded to the clinical status of the patient from whom the renal stones were obtained.

A stone sample was also sent for chemical quantitative analysis (Simon and Centzknow method). Pure struvite was defined as magnesium ammonium phosphate ± carbonate apatite, while a mixed struvite stone was defined as any amount of struvite with other stone compositions.

Post-operatively, complete blood count and renal function tests were repeated on the first post-operative day. Specific complications such as acute kidney injury and blood transfusion were noted separately, apart from classifying the patients according to the modified Clavien classification for PCNL.

Acute kidney injury was defined as an increase in post-operative creatinine of greater than 0.3 mg/dL within 48 h, according to the Acute Kidney Injury Network (AKIN) classification [33]. Furthermore, the duration of hospital stay and the need for re-admission were recorded. Patients were reviewed 1 month after the procedure and were placed on a regular follow-up schedule.

## 6. Statistical Analysis

Data was recorded on Microsoft Excel software, and analysis was performed using the SPSS v23 (IBM Corp., Armonk, NY, USA) software. The analysis of categorical variables was performed using the Chi-square test of association and the Student *t*-test or the Mann–Whitney U test for continuous variables. Following univariate analysis, binomial logistic regression was performed on statistically significant variables.

## 7. Conclusions

Stone culture is strongly associated with infective complications following PCNL. The presence of risk factors such as female gender, chronic kidney disease, bladder urine culture, stone size, a hypodense core on CT bone window, and a low CROES score are crucial in identifying patients with infected renal calculi, as they require vigilant post-operative monitoring. Mid-stream urine culture is associated with infected renal calculi and is a potential representation of the organism residing in the renal calculi. The occurrence of infective complications despite pre-operative antibiotic use may be attributed to multidrug-resistant organisms within infected stones. Stone culture and sensitivity testing is invaluable in guiding antibiotic therapy in patients presenting with urosepsis following PCNL for suspected infection stones and poor responses to empirical antibiotics. The only practical limitation of stone culture is the time required for the growth of organisms and the detection of antibiotic sensitivity, which reduces its clinical efficiency. Prior mid-stream urine culture can be used as an aid, if not a substitute, for choosing antibiotic therapies in the event of an infectious complication following PCNL in the absence or unavailability of stone culture.

## Figures and Tables

**Table 1:** Patient variables in comparison with stone culture—Univariate analysis.

Parameters	Stone Culture		*p*-Value
	Positive (*n* = 16)	Negative (*n* = 110)	
Age (Years)	53.50 ± 14.78	49.83 ± 11.88	0.355 ^1^
Age			0.419 ^2^
20–29 Years	0 (0.0%)	4 (3.6%)	
30–39 Years	4 (25.0%)	18 (16.4%)	
40–49 Years	3 (18.8%)	32 (29.1%)	
50–59 Years	4 (25.0%)	34 (30.9%)	
60–69 Years	2 (12.5%)	16 (14.5%)	
70–79 Years	3 (18.8%)	6 (5.5%)	
Gender ***			0.046 ^3^
Male	7 (43.8%)	76 (69.1%)	
Female	9 (56.2%)	34 (30.9%)	
Comorbidity: T2DM (Yes)	6 (37.5%)	29 (26.4%)	0.378 ^2^
Comorbidity: HTN (Yes)	6 (37.5%)	46 (41.8%)	0.743 ^3^
Comorbidity: IHD (Yes)	1 (6.2%)	10 (9.1%)	1.000 ^2^
Comorbidity: CKD 3 or Above(Yes) ***	5 (31.2%)	4 (3.6%)	0.002 ^2^
BMI (kg/m^2^)	24.98 ± 6.07	24.90 ± 4.37	0.959 ^1^
Recent Urological Intervention (Yes)	8 (50.0%)	30 (27.3%)	0.082 ^2^
Urine Culture (Pre-Operative) (Positive) ***	9 (56.2%)	11 (10.0%)	<0.001 ^2^
Haemoglobin (g/dL) (Pre-Operative) ***	10.64 ± 1.27	13.01 ± 2.08	<0.001 ^1^
Total Leukocyte Count (/mm^3^) (Pre-Operative)	8.85 ± 3.39	8.21 ± 2.12	0.644 ^4^
S. Creatinine (mg/dL) (Pre-Operative) ***	1.92 ± 1.53	1.14 ± 0.74	0.038 ^4^
S. Calcium (mg/dL) (Pre-Operative)	9.26 ± 0.52	9.40 ± 0.51	0.247 ^4^
S. Phosphorus (mg/dL) (Pre-Operative)	3.89 ± 0.77	3.57 ± 0.62	0.125 ^1^
Uric Acid (mg/dL) (Pre-Operative)	4.98 ± 1.27	5.26 ± 1.60	0.436 ^1^
Urine Random Calcium	6.78 ± 5.94	7.36 ± 5.87	0.608 ^4^
Urine Random Phosphorous	23.06 ± 15.09	26.18 ± 20.31	0.778 ^4^
Urine Random Magnesium	2.98 ± 1.37	3.95 ± 2.86	0.354 ^4^
Urine Random Uric Acid	20.14 ± 11.74	30.21 ± 22.47	0.132 ^4^
DJ Stent (Pre-Operative) (Yes)	4 (25.0%)	11 (10.0%)	0.099 ^2^
Catheter (Pre-Operative) (Yes)	2 (12.5%)	2 (1.8%)	0.078 ^2^

*** Significant at *p* < 0.05, ^1^: *t*-test, ^2^: Chi-squared test, ^3^: Wilcoxon–Mann–Whitney U test, and ^4^: Fisher’s exact test.

**Table 2:** Stone variables in comparison with stone cultures—Univariate analysis.

Parameters	Stone Culture		*p*-Value
	Positive (*n* = 16)	Negative (*n* = 110)	
Staghorn (Rassweiler Classification)			0.301 ^2^
No	9 (56.2%)	67 (60.9%)	
Borderline	1 (6.2%)	17 (15.5%)	
Partial	1 (6.2%)	12 (10.9%)	
Complete	5 (31.2%)	14 (12.7%)	
Staghorn (Mishra Classification) ***			<0.001 ^2^
1	6 (37.5%)	77 (70.0%)	
2a	1 (6.2%)	22 (20.0%)	
2b	5 (31.2%)	8 (7.3%)	
3	4 (25.0%)	3 (2.7%)	
Stone Analysis: Calcium (Yes)	15 (93.8%)	94 (85.5%)	0.695 ^2^
Stone Analysis: Magnesium (Yes) ***	5 (31.2%)	9 (8.2%)	0.017 ^2^
Stone Analysis: Uric Acid (Yes)	7 (43.8%)	46 (41.8%)	0.884 ^3^
Stone Analysis: Ammonia (Yes) ***	8 (50.0%)	19 (17.3%)	0.006 ^2^
Stone Analysis: Oxalate (Yes)	15 (93.8%)	92 (83.6%)	0.463 ^2^
Stone Analysis: Phosphate (Yes)	12 (75.0%)	78 (70.9%)	1.000 ^2^
Stone Analysis: Carbonate (Yes)	1 (6.2%)	2 (1.8%)	0.337 ^2^
Stone Side			0.838 ^3^
Left	8 (50.0%)	58 (52.7%)	
Right	8 (50.0%)	52 (47.3%)	
Stone Size (mm^2^)	753.94 ± 649.23	550.45 ± 740.76	0.147 ^4^
Stone Volume (mm^3^) ***	11,020.24 ± 11,795.32	4915.92 ± 7187.62	0.012 ^4^
Pelvis Stone (Yes)	13 (81.2%)	81 (73.6%)	0.759 ^2^
Upper-Calyx Stone (Yes)	5 (31.2%)	33 (30.0%)	1.000 ^2^
Middle-Calyx Stone (Yes)	8 (50.0%)	34 (30.9%)	0.130 ^3^
Lower-Calyx Stone (Yes)	13 (81.2%)	62 (56.4%)	0.058 ^3^
Severity of Hydronephrosis			0.103 ^2^
No	6 (37.5%)	32 (29.1%)	
Mild	4 (25.0%)	47 (42.7%)	
Moderate	4 (25.0%)	29 (26.4%)	
Gross	2 (12.5%)	2 (1.8%)	
Number of Stones	2.19 ± 1.52	2.32 ± 2.05	0.839 ^4^
Hounsfield Unit	947.75 ± 293.10	999.88 ± 331.43	0.521 ^1^
Tract Length	100.06 ± 17.90	99.77 ± 13.65	0.951 ^1^
Guy’s Score			0.145 ^2^
1	4 (25.0%)	36 (32.7%)	
2	6 (37.5%)	37 (33.6%)	
3	1 (6.2%)	24 (21.8%)	
4	5 (31.2%)	13 (11.8%)	
STONE Score	7.75 ± 2.02	7.21 ± 1.50	0.317 ^4^
CROES Score ***	156.44 ± 47.73	188.62 ± 57.82	0.023 ^1^

*** Significant at *p* < 0.05, ^1^: *t*-test, ^2^: Chi-squared test, ^3^: Wilcoxon–Mann–Whitney U test, and ^4^: Fisher’s exact test.

**Table 3:** Intra-operative variables in comparison with stone cultures—Univariate analysis.

Parameters	Stone Culture		*p*-Value
	Positive (n = 16)	Negative (n = 110)	
Operative Time (Minutes)	113.12 ± 61.72	85.32 ± 33.31	0.074 ^4^
Energy Source ***			0.028 ^2^
Lithotripter	15 (93.8%)	68 (61.8%)	
TFL	1 (6.2%)	41 (37.3%)	
Holmium	0 (0.0%)	1 (0.9%)	
Site of Puncture			0.816 ^2^
Supracostal	5 (31.2%)	26 (23.6%)	
Infracostal	10 (62.5%)	76 (69.1%)	
Supracostal/Infracostal	1 (6.2%)	8 (7.3%)	
Number of Punctures			1.000 ^2^
1	15 (93.8%)	97 (88.2%)	
2	1 (6.2%)	13 (11.8%)	
Upper-Calyx Puncture (Yes)	6 (37.5%)	41 (37.3%)	0.986 ^3^
Middle-Calyx Puncture (Yes)	7 (43.8%)	22 (20.0%)	0.053 ^2^
Lower-Calyx Puncture (Yes)	5 (31.2%)	57 (51.8%)	0.124 ^3^
Multiple Calyx Punctures (Yes)	1 (6.2%)	10 (9.1%)	1.000 ^2^
Amplatz Size ***	25.31 ± 4.33	22.65 ± 5.59	0.045 ^4^
Type of PCNL ***			0.037 ^3^
Standard	13 (81.2%)	59 (53.6%)	
Mini	3 (18.8%)	51 (46.4%)	
Residual Fragments (Yes) ***	6 (37.5%)	11 (10.0%)	0.009 ^2^
DJ Stent (Yes)	15 (93.8%)	105 (95.5%)	0.565 ^2^
Percutaneous Nephrostomy (Yes)	1 (6.2%)	4 (3.6%)	0.499 ^2^
Hypotension (Intra-Operative) (Yes)	7 (43.8%)	24 (21.8%)	0.068 ^2^

*** Significant at *p* < 0.05, ^2^: Chi-squared Test, ^3^: Wilcoxon–Mann–Whitney U test, and ^4^: Fisher’s exact test.

**Table 4:** Post-operative variables in the comparison of stone cultures with univariate analysis.

Parameters	Stone Culture		*p*-Value
	Positive (*n* = 16)	Negative (*n* = 110)	
Fever (Yes) ***	16 (100.0%)	15 (13.6%)	<0.001 ^2^
SIRS (Yes) ***	15 (93.8%)	15 (13.6%)	<0.001 ^2^
qSOFA Score ***	0.69 ± 0.60	0.31 ± 0.50	0.008 ^4^
qSOFA (Sepsis) (Yes)	1 (6.2%)	2 (1.8%)	0.337 ^2^
NEWS Score ***	6.62 ± 1.20	2.15 ± 2.02	<0.001 ^4^
NEWS Clinical Risk (Sepsis) ***			<0.001 ^2^
Low	0 (0.0%)	97 (88.2%)	
Medium	7 (43.8%)	7 (6.4%)	
High	9 (56.2%)	6 (5.5%)	
S. Creatinine (mg/dL) (Post-Operative) ***	2.08 ± 1.39	1.23 ± 0.65	<0.001 ^4^
Haemoglobin (g/dL) (Post-Operative) ***	10.01 ± 1.47	12.18 ± 2.09	<0.001 ^1^
Total Leukocyte Count (/mm^3^) (Post-Operative) ***	16.73 ± 6.72	11.79 ± 3.91	<0.001 ^4^
Hospital Stay (Days) ***	5.31 ± 1.85	3.18 ± 1.02	<0.001 ^4^
Re-Admission (Yes)	0 (0.0%)	8 (7.3%)	0.595 ^2^
PCN-Site Infection (Yes)	2 (12.5%)	6 (5.5%)	0.268 ^2^
Follow-Up: Fever (Yes) ***	16 (100.0%)	15 (13.6%)	<0.001 ^2^
Modified Clavien–Dindo Classification ***			<0.001 ^2^
None	0 (0.0%)	64 (58.2%)	
1	0 (0.0%)	18 (16.4%)	
2	5 (31.2%)	17 (15.5%)	
3A	11 (68.8%)	8 (7.3%)	
3B	0 (0.0%)	1 (0.9%)	
4A	0 (0.0%)	2 (1.8%)	
Acute Kidney Injury (Post-Operative) (Yes) ***	6 (37.5%)	14 (12.7%)	0.021 ^2^
Haemoglobin Drop (g/dL)	0.76 ± 0.77	0.93 ± 0.96	0.568 ^4^
Blood Transfusion (Yes)	2 (12.5%)	4 (3.6%)	0.168 ^2^

*** Significant at *p* < 0.05, ^1^: *t*-test, ^2^: Chi-squared test, and ^4^: Fisher’s exact test.

**Table 5:** Antibiotic sensitivity of various isolated organisms in renal stone cultures.

Parameters	Stone Culture Organism						
	*E. coli* (n = 5)	*Klebsiella pneumoniae* (n = 3)	*E. faecalis* (n = 2)	*Pseudomonas aeruginosa* (n = 1)	*Proteus mirabilis* (n = 3)	*Salmonella* spp. (n = 1)	*Staphylococcus Aureus* (n = 1)
AST: Ceftazidime							
Sensitive	NT	NT	NT	1 (100.0%)	1 (100.0%)	NT	0 (0.0%)
Intermediate	NT	NT	NT	0 (0.0%)	0 (0.0%)	NT	0 (0.0%)
Resistant	NT	NT	NT	0 (0.0%)	0 (0.0%)	NT	1 (100.0%)
AST: Ciprofloxacin/Ofloxacin							
Sensitive	0 (0.0%)	0 (0.0%)	0 (0.0%)	1 (100.0%)	0 (0.0%)	0 (0.0%)	0 (0.0%)
Intermediate	0 (0.0%)	0 (0.0%)	0 (0.0%)	0 (0.0%)	1 (33.3%)	0 (0.0%)	0 (0.0%)
Resistant	5 (100.0%)	3 (100.0%)	2 (100.0%)	0 (0.0%)	2 (66.7%)	1 (100.0%)	1 (100.0%)
AST: Cefoperazone–Sulbactam							
Sensitive	3 (60.0%)	1 (50.0%)	NT	NT	1 (100.0%)	NT	NT
Intermediate	0 (0.0%)	0 (0.0%)	NT	NT	0 (0.0%)	NT	NT
Resistant	2 (40.0%)	1 (50.0%)	NT	NT	0 (0.0%)	NT	NT
AST: Cloxacillin							
Sensitive	NT	NT	NT	NT	NT	NT	1 (100.0%)
Intermediate	NT	NT	NT	NT	NT	NT	0 (0.0%)
Resistant	NT	NT	NT	NT	NT	NT	0 (0.0%)
AST: Trimethoprim/Sulphamethoxazole							
Sensitive	4 (80.0%)	1 (33.3%)	NT	NT	0 (0.0%)	1 (100.0%)	1 (100.0%)
Intermediate	0 (0.0%)	0 (0.0%)	NT	NT	0 (0.0%)	0 (0.0%)	0 (0.0%)
Resistant	1 (20.0%)	2 (66.7%)	NT	NT	2 (100.0%)	0 (0.0%)	0 (0.0%)
AST: Erythromycin							
Sensitive	NT	NT	0 (0.0%)	NT	NT	NT	1 (100.0%)
Intermediate	NT	NT	0 (0.0%)	NT	NT	NT	0 (0.0%)
Resistant	NT	NT	1 (100.0%)	NT	NT	NT	0 (0.0%)
AST: Gentamicin							
Sensitive	3 (60.0%)	0 (0.0%)	2 (100.0%)	NT	1 (50.0%)	NT	1 (100.0%)
Intermediate	0 (0.0%)	0 (0.0%)	0 (0.0%)	NT	0 (0.0%)	NT	0 (0.0%)
Resistant	2 (40.0%)	3 (100.0%)	0 (0.0%)	NT	1 (50.0%)	NT	0 (0.0%)
AST: Tetracycline/Doxycycline							
Sensitive	NT	NT	0 (0.0%)	NT	NT	NT	1 (100.0%)
Intermediate	NT	NT	0 (0.0%)	NT	NT	NT	0 (0.0%)
Resistant	NT	NT	2 (100.0%)	NT	NT	NT	0 (0.0%)
AST: Clindamycin							
Sensitive	NT	NT	NT	NT	NT	NT	1 (100.0%)
Intermediate	NT	NT	NT	NT	NT	NT	0 (0.0%)
Resistant	NT	NT	NT	NT	NT	NT	0 (0.0%)
AST: Amikacin							
Sensitive	4 (80.0%)	1 (33.3%)	NT	NT	1 (50.0%)	NT	NT
Intermediate	1 (20.0%)	0 (0.0%)	NT	NT	0 (0.0%)	NT	NT
Resistant	0 (0.0%)	2 (66.7%)	NT	NT	1 (50.0%)	NT	NT
AST: Cefotaxime/Ceftriaxone							
Sensitive	0 (0.0%)	0 (0.0%)	NT	NT	1 (50.0%)	1 (100.0%)	NT
Intermediate	0 (0.0%)	0 (0.0%)	NT	NT	0 (0.0%)	0 (0.0%)	NT
Resistant	5 (100.0%)	3 (100.0%)	NT	NT	1 (50.0%)	0 (0.0%)	NT
AST: Cefuroxime							
Sensitive	0 (0.0%)	0 (0.0%)	NT	NT	1 (50.0%)	NT	NT
Intermediate	0 (0.0%)	0 (0.0%)	NT	NT	0 (0.0%)	NT	NT
Resistant	5 (100.0%)	3 (100.0%)	NT	NT	1 (50.0%)	NT	NT
AST: Amoxicillin–Clavulanic Acid							
Sensitive	0 (0.0%)	0 (0.0%)	NT	NT	1 (50.0%)	NT	NT
Intermediate	1 (20.0%)	1 (33.3%)	NT	NT	1 (50.0%)	NT	NT
Resistant	4 (80.0%)	2 (66.7%)	NT	NT	0 (0.0%)	NT	NT
AST: Cefipime/Cefpirome							
Sensitive	0 (0.0%)	0 (0.0%)	NT	NT	0 (0.0%)	NT	NT
Intermediate	0 (0.0%)	0 (0.0%)	NT	NT	0 (0.0%)	NT	NT
Resistant	5 (100.0%)	3 (100.0%)	NT	NT	1 (100.0%)	NT	NT
AST: Imipenem							
Sensitive	5 (100.0%)	1 (33.3%)	NT	NT	NT	NT	NT
Intermediate	0 (0.0%)	1 (33.3%)	NT	NT	NT	NT	NT
Resistant	0 (0.0%)	1 (33.3%)	NT	NT	NT	NT	NT
AST: Piperacillin–Tazobactam							
Sensitive	2 (40.0%)	1 (33.3%)	NT	NT	1 (100.0%)	NT	NT
Intermediate	0 (0.0%)	0 (0.0%)	NT	NT	0 (0.0%)	NT	NT
Resistant	3 (60.0%)	2 (66.7%)	NT	NT	0 (0.0%)	NT	NT
AST: Tigecycline							
Sensitive	1 (100.0%)	1 (100.0%)	NT	NT	NT	NT	NT
Intermediate	0 (0.0%)	0 (0.0%)	NT	NT	NT	NT	NT
Resistant	0 (0.0%)	0 (0.0%)	NT	NT	NT	NT	NT
AST: Meropenem							
Sensitive	3 (100.0%)	1 (33.3%)	NT	NT	1 (100.0%)	NT	NT
Intermediate	0 (0.0%)	0 (0.0%)	NT	NT	0 (0.0%)	NT	NT
Resistant	0 (0.0%)	2 (66.7%)	NT	NT	0 (0.0%)	NT	NT
AST: Benzylpenicillin							
Sensitive	NT	NT	2 (100.0%)	NT	NT	NT	NT
Intermediate	NT	NT	0 (0.0%)	NT	NT	NT	NT
Resistant	NT	NT	0 (0.0%)	NT	NT	NT	NT

Abbreviations: AST—Antibiotic Sensitivity Test; NT—not tested.

**Table 6:** Regression analysis with individual variables—Infectious complications and clinical indicators (Univariate).

Dependent: Stone Culture		Negative	Positive	OR (Univariable)
Fever	No	95 (100.0)	0 (0.0)	911,504,457.93 (0.00–NA, *p* = 0.991)
	Yes	15 (48.4)	16 (51.6)	
SIRS	No	95 (99.0)	1 (1.0)	95.00 (17.37–1781.76, *p* = 0.001)
	Yes	15 (50.0)	15 (50.0)	
qSOFA Score	0	78 (92.9)	6 (7.1)	3.24 (1.31–8.39, *p* = 0.011)
	1	30 (76.9)	9 (23.1)	
	2	2 (66.7)	1 (33.3)	
NEWS Score	Mean (SD)	2.1 (2.0)	6.6 (1.2)	2.43 (1.77–3.74, *p* = 0.001)
NEWS Clinical Risk (Sepsis)	Low	97 (100.0)	0 (0.0)	-
	Medium	7 (50.0)	7 (50.0)	854,535,429.30 (0.00–NA, *p* = 0.991)
	High	6 (40.0)	9 (60.0)	1,281,803,143.96 (0.00–NA, *p* = 0.991)

**Table 7:** Univariate logistic regression analysis of statistically significant pre-operative predictors on univariate analysis for stone culture positivity.

Predictors	*p*-Value	Odds Ratio	95% Confidence Interval	
			Lower	Upper
Gender Female–Male	0.053	2.87	0.99	8.35
CKD Yes–No	0.001	12.04	2.81	51.56
Urine C/S Pre-Operative Positive–Negative	0.001	11.57	3.60	37.20
Stone Volume (mm^3^)	0.014	1.0001	1.00	1.00
CROES Score	0.041	0.99	0.98	1.00

**Table 8:** Studies correlating stone culture with sepsis following PCNL.

S. No	Author (Ref)	Type of Study	Association Variables	*p*-Value
1	Ramaraju et al. [26]	Prospective	Stone culture and SIRS	0.003
2	Silvani et al. [27]	Prospective	Stone culture and infectious complications (Fever/SIRS)	0.008
3	Mariappan et al. [8]	Prospective	Stone culture and SIRS	0.0009
4	Mishra et al. [21]	Prospective	Stone culture and SIRS	0.0001
5	Sen et al. [28]	Retrospective	Stone culture and SIRS	<0.001
6	Chen et al. [29]	Prospective	Stone culture and qSOFA	<0.001
7	Danilovic et al. [30]	Retrospective	Stone culture and SOFA	0.36
8	Present study	Prospective Cohort Study	Stone culture and fever	<0.001
			Stone culture and SIRS	<0.001
			Stone culture and qSOFA (Sepsis ≥ 2)	0.337
			Stone culture and NEWS	<0.001
			Stone culture and NEWS clinical risk (sepsis)	<0.001

## Data Availability

The original contributions presented in this study are included in the article. Further inquiries can be directed to the corresponding author.

## Abbreviations

The following abbreviations are used in this manuscript:

PCNL	Percutaneous Nephrolithotomy
SIRS	Systemic Inflammatory Response Syndrome
CROES	Clinical Research Office of Endourological Society
AKI	Acute Kidney Injury
SPSS	Statistical Package for Social Sciences
IEC	Institutional Ethics Committee
CTRI	Clinical Trials Registry of India
MSUC	Mid-Stream Urine Culture
NCCT	Non-Contrast Computed Tomography
KUB	Kidney Ureter Bladder
AKIN	Acute Kidney Injury Network
SCP	Stone Culture-Positive
SCN	Stone Culture-Negative
NEWS	National Early Warning Score
qSOFA	Quick-Sequential Organ Failure Assessment
CKD	Chronic Kidney Disease
UTI	Urinary Tract Infection
PCN	Percutaneous Nephrostomy
